# Vanilla bisquits and lobola bridewealth: parallel discourses on early pregnancy and schooling in rural Zambia

**DOI:** 10.1186/s12889-020-09555-y

**Published:** 2020-10-01

**Authors:** Astrid Blystad, Karen Marie Moland, Ecloss Munsaka, Ingvild Sandøy, Joseph Zulu

**Affiliations:** 1 CISMAC (Centre for Intervention Science in Maternal and Child Health), Bergen, Norway; 2 Centre for International Health, Department of Global Public Health and Primary Care, University of Bergen, Bergen, Norway; 3 School of Education, University of Zambia, Lusaka, Zambia; 4 School of Public Health, University of Zambia, Lusaka, Zambia

**Keywords:** Adolescent pregnancy, School dropout, National-local discourse, Zambia

## Abstract

**Background:**

Adolescent pregnancy is a complex socio-economic phenomenon ranking high on the global health policy agenda. Early childbearing is associated with early marriage and school drop-out, and is defined as a problem to the health and development of girls. This paper reports from formative research. The formative research aimed to explore socio-cultural and structural dynamics at work behind early pregnancy and school drop out in rural Zambia. The study findings have been used to inform a school based intervention to reduce early pregnancy (RISE: ‘Research Initiative to Support the Empowerment of Girls’). Theoretically the study is informed by social constructionism.

**Methods:**

A qualitative approach was employed. Semi-structured qualitative interviews (61) and focus group discussions (7) were carried out with girls (in and out of school), boys, parents, teachers, health workers and community- and district leaders in 2014–15. Systematic text condensation was drawn upon in the analysis of the material.

**Results:**

The study findings indicate that the official Zambian discourse that presents early pregnancy as a serious challenge and schooling as the prime way to confront the problem enjoy substantial support at community levels. However, a parallel discourse on fertility, early marriage and childbearing as social and economic security surfaced and was articulated by the same study participants. The latter contrasting discourse questioned schooling as the only solution to secure a girl’s future arguing that there are many reasons why early pregnancy may emerge as rational.

**Conclusions:**

Grasping the complexity of local discourse is vital in planning health interventions. The present study revealed that although delayed child bearing and schooling among girls enjoyed high status and legitimacy in the study area, the social and economic context worked to reward early marriage. Interventions to reduce early pregnancies in rural Zambian communities need to fundamentally address the material constraints that condition and reinforce a culture of early childbearing.

## Background

Early childbearing has during the last decades received increasing attention as a threat to the health and development of girls, and an increasing body of public health research documents the health risks of early pregnancy [1–3]. Early pregnancy is intimately connected with early marriage and school drop-out producing dynamics perceived to have substantial negative implications for girls’ life courses [4]. Sustained high levels of pregnancy and birth rates among adolescents are found to be particularly dominant in sub-Saharan Africa [1], and are obstacles that prevent young girls and women from fulfilling their potential. Gender equality and girls’ right to reproductive health lie at the heart of diverse initiatives to fight adolescent pregnancy [1, 5]. The WHO guidelines on preventing early pregnancy and poor reproductive outcomes among adolescents in developing countries spell out a comprehensive multi-sectoral approach to combat early pregnancy through marriage law reform. It includes interventions targeting community norms pertaining to the timing of marriage, the empowerment of girls and increasing educational opportunities. Through such initiatives countries in sub-Saharan Africa are supported to develop strategies to decrease adolescent pregnancy and school dropout [1].

Another and partly interlinked body of literature focuses on the dynamics at work behind early pregnancy and motherhood [2–4, 6, 7]. The literature reveals that dynamics leading to adolescent pregnancy are complex and entangled, and commonly highlights a series of interlinked socio-cultural and structural factors. Customary early marriage, the bride-wealth institution and gender norms have been pointed out as central to the understanding of early pregnancy [8–12]. A number of ethnographic sources have moreover illustrated a substantial customary value placed on child bearing and fertility in sub-Saharan African societies [13–20], and have explored the dynamics behind the timing of pregnancy [9, 13, 21–25]. Explanations of early pregnancy focusing on ‘culture’ or ‘cultural disintegration’ have been coupled with economic- or structural explanations addressing contexts of poverty and marginalization, a lack of proper sexual- and reproductive health education and poor access to and appropriateness of public health services for the young [2, 4, 7, 12].

There are important examples in public health publications that deal with early pregnancy in nuanced ways, paying attention to early pregnancy as desired and having social benefit, such as the works of McCleary-Sills et al. [26]. Nonetheless, there is a tendency of approaching the ‘problem of teenage pregnancy’ from a relatively narrow health-oriented angle in public health. This has importantly been problematized in the broader literature. Preston-Whyte has for example discussed the massive international and national policy pressure to fight early childbearing in the southern African region. Through fine-grained ethnographic studies she has focused on the dynamics of fertility and early premarital pregnancy from South Africa [20, 23–25], and has demonstrated how many teenagers and parents perceive premarital early pregnancy as a rational strategy to achieve a set of important goals. Preston-Whyte and others have argued that ‘teenage pregnancy’ cannot automatically and unequivocally be represented as an individual or social problem. The studies of MacLeod [10] and Mkahwanazi [27, 28] investigate the historical, political and social power relations and discursive practices that construct adolescent pregnancy as harmful. The authors argue that in view of socio-economic conditions and normative patterns found throughout the sub-Saharan region, early marriage and pregnancy have often been desired and are perceived as a rational choice.

Jewkes et al. [22] have pointed out that in exploring factors behind early pregnancy, one will often encounter strong co-existing discourses. In this paper we explore prevailing parallel discourses on early pregnancy, early marriage and school dropout as encountered among community members in rural Zambia. The main aim of this formative study was to expand the knowledge base on socio-cultural and structural dynamics at work behind early pregnancy and school dropout, including perceptions about early pregnancy as a social phenomenon. The study findings have been used to inform school based intervention research targeting early pregnancy in rural Zambia (‘Research Initiative to Support the Empowerment of Girls’).

### The study context

In Zambia, childbearing starts early and teenage pregnancy is common, particularly in rural areas. According to the latest Demographic and Health Survey (DHS) of 2013/14, one third of Zambian women have given birth by the age of 18 and more than half by the age of 20 [29]. Adolescent pregnancies are considerably higher in rural than urban areas (30% vs 17%), and in the lowest wealth quintile compared to the highest (37% vs 6%). The age of birth giving is moreover associated with education, and the age at first birth increases with education. Women with no education give birth around 2 years earlier than women with secondary education. Education is stated as a right and all school age children are expected to go to school in Zambia [30], but school enrolment and drop-outs are highly unequal between boys and girls and between rural and urban areas.

Early pregnancy is closely related to early marriage. The legal age of marriage is 21 unless parents provide consent to marriage before this age [31]. Nevertheless, the DHS 2013/14 reports that 45% of women in the age group 25–49 were married by the age of 18 [29]. Early marriage and early pregnancy are associated with poverty, and the scale of adolescent marriage and childbearing is perceived to be a societal challenge of substantial proportions in Zambia. Poverty is again particularly widespread in rural areas where about 70% of the population is categorised as poor [29].

Zambia is by constitution a Christian Nation, and the Christian discourse tends to permeate public debate and promote religious values that may constrain people from accessing sexual and reproductive health (SRH) services including family planning [32]. Comprehensive Sexuality Education was nonetheless introduced in the country in 2014 [33], but preliminary evidence indicates implementation challenges and limited support from parents and the general public.

The government has defined schooling of girls as a major strategy to prevent teenage pregnancy and as a condition for girls’ social and economic development [34, 35]. A prime challenge is however that increased schooling does not necessarily lead to employment. Waged labour is scarce in rural Zambia and subsistence farming is the most common activity in rural areas. Beyond employment in the public sector as educated teachers and health workers or in private ranching, farming or mining companies, relatively stable employment opportunities are limited. In recent years public employment has become more cumbersome than earlier, with large numbers of educated teachers remaining unemployed for years before they get their first job. Small scale business and petty trading remain the most viable paid opportunities for many.

In the effort to address teenage pregnancies and early marriage, Zambia has moreover committed to a number of international and national policies and declarations. Among others it has ratified the UN Convention on the Rights of the Child and the African Charter on Human and People’s Rights on the Rights of Women. In line with the Sustainable Development Goals agenda, the country has committed to Goal 5 on gender equality to eliminate child, early and forced marriage by 2030 (target 5.3). In relation to Goal 3 on health and wellbeing (target 3.7) Zambia has moreover committed to ensure universal access to SRH services [36]. Access to sexual and reproductive health services (SRH) is nonetheless found to be very limited and again unequally distributed with a huge urban bias. The unmet need for contraceptives is high, especially among teenagers aged 15–19 years where it is estimated at 25% [1].

This paper is based on qualitative formative research to inform the development of interventions within the ‘Research Initiative to Support the Empowerment of Girls’ (RISE) [30]. RISE is designed as a cluster randomised trial (2015–2020) and aims to measure the effectiveness of a school based intervention targeting early childbearing, marriage and school drop-out among girls in rural Zambia. The RISE project is run by the University of Zambia in collaboration with the University of Bergen, Norway. It is anchored within the Centre for Intervention Science in Maternal and Child Health (CISMAC), an international consortium performing research to develop, test and implement interventions to improve maternal and child health outcomes in low and middle income countries (https://cismac.uib.no/). Nichter et al. [37] call for qualitative formative research to develop, monitor or critically assess interventions in public health. A thorough mapping of the perceived dynamics surrounding early pregnancy and school dropout was deemed important for the development of the RISE intervention study [2, 20, 21]. The main research question asked in this formative project was: How does official discourse on early pregnancy as a problem and schooling of girls as solution, play out and articulate with local understanding of early pregnancy, early marriage and school dropout in rural Zambia?

## Methods

### Theoretical approach

In our attempt to make sense of prevailing discourses on early pregnancy, we draw upon social constructionist approaches. Building on Foucault’s notion of truth being created discursively, we define discourse as “a particular way of talking about and understanding the world (or an aspect of the world)” [38]. Social constructionist approaches provide researchers with a critical approach to taken-for-granted knowledge, and bring attention to the historical and cultural specificity of knowledge and to the link between knowledge and social processes. Social constructionism emphasizes that ways of talking do not neutrally reflect our world, identities and social relations, but rather play an active role in creating and continuously changing them. The latter point indicates the manner in which diverse social understandings or meaning making of the world are linked to social action. In the present study a social constructionist approach informed the questions asked and the analysis and discussion of the study findings.

### Study setting

The present study was carried out in five of the districts selected for the RISE study and included Mazabuka, Chikankata and Monze located in Southern Province and Chibombo and Kapiri Mposhi in Central Province. The districts are located some 95 km or more from the capital Lusaka, and have relatively high rates of early pregnancy compared to other rural districts in Zambia. According to the 2010 census, estimates of the percentages of girls ‘ever given birth’ in the study districts was 4% among 15 year olds, 9.5% among 16 year olds, 22% among 17 year olds and 35% among 18 year olds [39]. Southern Province is largely inhabited by Tonga speaking people while Central Province is predominately Bemba-speaking. Tonga speaking people are cattle keeping pastoralists. Both Southern and Central provinces are otherwise characterized by subsistence farming with maize as main crop.

### Data collection

The data collection process was divided into two phases; Phase I was carried out in November and December 2014 exploring perceptions of early pregnancy and early marriage and the dynamics behind the high adolescent pregnancy rates. Phase II took place in February and March 2015 following up on central knowledge gaps identified during phase I, and investigated community reflections on a set of interventions developed partly on the basis of findings from Phase I. This paper reports and discusses research findings from Phase I and II related to dynamics behind early pregnancy, early marriage and school drop-out as expressed by the study informants.

A total of 61 semi-structured interviews and seven (7) focus group discussions (with girls 13–16 years old) were conducted during the two study phases. The main categories of informants were girls (in−/out-of-school, married/non-married, had been pregnant/had not been pregnant), boys, parents, teachers, health workers, community leaders and district level officials. Community leaders assisted in the recruitment of the informants. The general qualitative research principle of data saturation was employed in the recruitment process.

Two of the co-authors (EM, JZ) actively engaged in the data collection process together with research assistants under close supervision. Interview- and topic guides were employed during the interviews and were developed for this particular study. They have not been used or published elsewhere. The guides contained open-ended questions developed for each of the main categories of informants. Both the interviews and the focus group discussions allowed the study participants to reply freely and at length. English language versions of all the guides are uploaded as supplementary files (Additional files 1, 2, 3, 4, 5, 6, 7, 8).

Phase I included questions such as: “Please tell me about early pregnancy in this area? What do you think are the reasons behind the increasing numbers of girls who become pregnant early? In your opinion what is the relationship between early pregnancy and school drop out? To what extent do you think early pregnancy may be desired by girls/boys/parents?” Interviews during the Phase II contained questions like: “How do you think the payment of school fees for girls (only) would be perceived by the girls/boys/ parents/community leaders? How do you think a small sum of pocket money provided to girls would be perceived by the girls/boys/parents/community leaders? How do you think a youth club, containing sexual and reproductive health education for the empowerment of girls and boys, will be perceived by the community?” The topic guides employed during the focus groups (among girls) brought up the same general topics, but contained fewer questions than the interview guides to allow the discussion (among girls) to flow with as little interference from the moderator as possible. The interviews and discussions were carried out in the Tonga, Nyanja and Bemba languages. The content of the interviews and the particularities of the research process were discussed at the end of each working day, facilitating adjustment of the guides and approach during the course of the data collection period.

The study topic was in principle not perceived to be particularly sensitive, and informants willingly participated in the study and actively engaged in the discussions. As we will see below however, views that were not perceived to fit into the dominant discourse on early pregnancy were voiced further into the interviews and – as we shall return to below - were commonly addressed with reference to others’ and not ones’ own point of view or opinion. We encountered patterns reflecting two distinct major discourses, discourses that to quite some extent emerged as contrasting.

### Data analysis

All interviews were audio recorded, and the recorded material was transcribed verbatim. The interviews were translated to English. Particular attention was paid to a word by word translation in attempts to keep the translated transcripts as close as possible to the original recorded material. Continuous checks were made during the translation process. The transcriptions were entered into QSR NVIVO 10.

Following key principles of qualitative research, the analysis started during the fieldwork. During the post fieldwork phase ‘systematic text condensation’ [40], an explorative method of analysis was employed. The procedure followed the four analytical steps: 1) Total impression: During this first step, all transcripts were shared and reviewed among the co-authors - authors based in anthropology, education, political science, health science and epidemiology - in order for all to gain an impression of the full material. 2) Identifying and sorting meaning units – from themes to codes; The full review was followed by the coding of all the transcripts identifying and sorting out meaning units carried out separately by three of the co-authors (AB, KMM, JZ). A main task during this step was a search for consistencies and seeming inconsistencies both within individual transcripts, between transcripts within the same category of informants as well as a comparison across diverse categories of informants. 3) Condensation – from code to meaning; The codes were subsequently merged into larger categories and themes in processes of condensation into meaningful units. 4) Synthesizing. The categories and themes were finally synthesized into written text. A series of written drafts were during this final step shared and commented upon by all co-authors.

### Ethics

The focus of the study, the study objectives and the research ethical principles were presented before seeking informed consent from the study participants. For girls below 16 years of age, consent from parents and assent from the girls were obtained. Written consent / assent was obtained from all categories of study participants. All consent was written. Substantial care was taken to ensure confidentiality and anonymity throughout the data collection, analysis and write-up process.

## Results

The study findings strongly reflect the public discourse in Zambia which presents early pregnancy as a moral and development problem. But the findings simultaneously reveal how the officially endorsed discourse is permeated by an alternative and parallel discourse that constructs early pregnancy and marriage as a valued and necessary means to social and economic security for girls. Thus, the social construction of the problem at hand presented the researchers with two rather contrasting - and seemingly opposing - discourses on early pregnancy and marriage. The contrasting discourses emerged across different categories of informants; among school-girls and girls who were out of school, among girls with- and without children, as well as among the other main categories of informants like parents, teachers, health workers and community leaders. There was a tendency of teachers and community leaders to more powerfully endorse the policy emphasizing schooling of girls as decisive in efforts to postpone pregnancy and marriage. But even among these informants the topic of why early pregnancy at times would be a necessary, chosen or rational option, would be raised. Importantly, individual study participants would commonly offer differing views during the course of the interview. The contrasting discourses moreover emerged both during individual interviews and during focus group discussions.

### On early pregnancy, early marriage and school dropout

#### ‘I want you to learn’: the threats of early pregnancy to further schooling

The highly interlinked dynamics of early pregnancy, early marriage and school dropout was by all categories of informants phrased as a problem. A Community Volunteer said:

*My 14 year old child ran away to get married. 14 years old she left school…14 years! She was in grade seven, she passed properly to grade eight. Before they opened grade eight she fell pregnant! I brought her here, - ha! I bought her uniforms. ‘I will keep you, I will pay for someone to take care of the baby. I want you to learn. Look at me, your parent, I am poor. What made me poor is what? Not going to school! That is what contributed to me being poor. Now my child I want you to do what? Learn!’*

School dropout caused by early pregnancy and early marriage was said to be a problem of substantial scale in the area: A nurse stated; *I have seen a lot of dropouts. Some don’t want to go. They just take off their uniforms and say ‘I’m done*’ (Nurse, Health Centre). A District Education Board Secretary Planner similarly said: *It’s rampant. It’s all over, all the schools are affected. It’s there seriously, it’s there in every school*. Commenting on the scale of early marriage a Guidance and Counselling Teacher expressed with a sigh: *You go to the wedding and you find a grade seven pupil is the bride. We have these nearly every week.. It’s not just at our school.*

Early pregnancy, early marriage and school drop-out were in the interviews commonly talked about in one breath. Pregnancy was said to lead to marriage and school drop-out, while school dropouts similarly were said to lead to early pregnancy, − marriage taking place both before and after a pregnancy, and among girls both in and out of school: *Soon after (quitting school) you hear that she is pregnant, afterwards she is married. At times leaving school without reason - yes, without being pregnant she may stop (school)* (Nurse, Health Centre).

The large majority of the girls interviewed – girls both in and out of school – would explicitly state that they wished to continue schooling and thus did not wish to become pregnant at a young age. The wish was commonly expressed in terms of a desire to assist their families, a typical statement being: “*The beauty about school is helping your parents away from their suffering”* (FGD girls). Parents, community members and leaders would similarly strongly emphasize the importance of schooling for all children. A preference for boys’ education was, however, often mentioned in passing: “*Some families say that if I send a girl (to school)…, a girl might become pregnant and then disappoint me. Unlike a boy… So let me just sponsor a boy, a boy can continue”* (Guidance and Counselling Teacher). As we shall see below, despite the powerful emphasis on education, hindrances to educate children, and particularly to the schooling of girls, were perceived to be many and complex.

### On early pregnancy and poverty

#### ‘You have grown, we cannot continue staying with you’

Pregnancy and marriage in adolescence was often talked about as ‘bad’ or ‘wrong’ as it would lead to continued circles of poverty: *If a girl of 13 years is impregnated by a boy of 16 years, they are not ready to be parents. It will mean poverty. Early marriages and early pregnancies are perpetuating poverty* (Education Officer). At the same time, poverty was described as a driver of early pregnancy, early marriage and school dropout as illustrated in the following discussion among girls in a focus group (..marks a shift to a new group participant):

*The thing that makes young girls get married early is that some parents encourage early marriage, saying ‘get married so that we can find money, look at how we are suffering’. So that is what makes the girls get married early, they get encouraged. The parents say ‘you have grown, we cannot continue staying with you’… What makes young girls get married early is that in the village where they stay, they don’t live well, the parents are poor and they don’t have money to pay school fees for the girl. So when a boy comes and starts proposing to this girl she will not refuse. This is how girls get married* (FGD, girls).

A married girl similarly described hardships at home as a reason behind early marriages:

*Like us, young girls who get into marriages early, - it’s because we find problems from our parents. I saw how my mother used to struggle to find money for my uniforms and school fees – that is how I got ideas of getting married,- because of the hard life I had.*

The lack of sufficient resources to pay school fees was iterated by a number of informants. A Nurse from a health centre explained: *The only thing that makes people become like this (become pregnant and fall out of school) is the cost of education. Education cost is too high. A majority of the people are failing to reach those costs*. A mother similarly stated: *For others pregnancy is due to the fact that parents have nothing to offer them, they have no money for their children to go and learn at school, as much as they would want their children to get educated*.

It was expressed that in contexts of very limited resources, children would readily end up as assets in the fight against poverty: *You know with this poverty in the country, some people are taking advantage of their own children, trying to make them do things that will bring something to eat* (Nurse, Health Centre). The consequences of severe financial constraints emerged as an aspect of nearly every topic that was discussed in relation to early pregnancy, a constraint at times intertwined with locally embedded customs and institutions as we shall explore in the coming sections.

#### Lobola bridewealth: an avenue to secure an income

Bride-wealth, *lobola*, was a custom found to be strongly associated with early marriage, i.e. wealth through cattle transferred from a man to a woman’s or girls’ family at the time of marriage. In rural Zambia the bride-wealth customarily consists of five heads of cattle. However, as one teacher explained: *If they are poor and don’t have cows, they are charged money worthy of two cows* (Community Volunteer). *Lobola* was said to continue to provide an important source of income for many families with daughters, and numerous informants brought up the interconnections between *lobola* and school drop-out. A local leader observed: *Those who know the importance of education know that education is a person’s wealth. But some parents still pay more attention to cattle than to school* (Ward Councilor). A Community Volunteer similarly expressed: *Some know the importance school has, but what they respect is marriage in order to ‘eat lobola.’* The prospects of receiving *lobola* was explained to fuel an interest in marrying off daughters, and a consequent lack of interest in secondary schooling for their daughters. Some were said to exploit the inherent resource potential in *lobola: Some men have a lot of wealth, but he doesn’t want to send his children to school. Bringing just one animal to sell so that his child can go to school and he feels like he has lost a lot.* Informants’ of all categories emphasized the dilemmas generated by poverty as becoming tangible when having to assess the potential income from *lobola* against the costs of extended schooling for their daughters.

#### Girls’ desire for ‘vanilla’

The element of exchange inherent in sexual relations provided another example of a custom strongly associated with early pregnancy and school drop-out. Girls commonly received biscuits, a coke or some cash from boys, but with the expectation of providing something in return. The discussion between the interviewer and a group of girls in a Tonga community illustrates the point:

*Interviewer (I): How much can they (the boys/men) afford? Participant (P): Like 50 kwacha (Zambian monetary unit, approximately 8 USD). I: What about you? How much do you see or hear? P: If you get a boyfriend and he is working he can even give you up to K 500 (approx. 80 USD). I: Are there a lot of men here who are working? P: No, not here. They are not here. I: What about buying us stuff? What kinds of things do they buy us? P1: They just buy us sweets. P2: Biscuits and jiggies. P3: Vanilla biscuits* (FGD, girls).

It was clear from the interviews that commonly neither boys nor girls had substantial access to cash, although boys were said to have a somewhat easier access to petty trade and were thus often able to afford cheap sweets or biscuits. The desire for ‘vanilla’ biscuits was indeed a recurrent theme in the interviews, and emerged as a metaphor for young girls’ emerging material desires and wish to have a boyfriend. The temptation to accept a gift was dwelled upon by many adults informants, often in statements condemning the poor moral judgement of girls: *Some girls love money too much, and if they don’t have (money) they get ideas of sleeping with a man so that they can make some money. That is the reason why girls get pregnant* (Community Health Worker).

#### The value of marriage and childbearing: ‘All they think of is marriage and getting pregnant’

Informants emphasized that childbearing had substantial value in their communities. Although strong sentiment against early pregnancy and marriage was expressed across all categories of informants, an underlying tension between ideals of extended schooling and ideals of childbearing strongly emerged in the interviews and discussions. The latter preference was commonly not expressed as their own views and preferences but as the unacceptable ‘values of others’. The value of having children was moreover often made with reference to marriage. A Community Volunteer observed:

*Marriage is important to them (the girls), equally, having children is important. They stop school to go and get married. Maybe parents take them back to school, beat them. But upon reaching home the girl flees to get married. Yes, that is what they do. Marriage is very important to them.*

Commenting upon how difficult it is to stop early marriage and early pregnancy, another Community Volunteer explained: *Getting married early and early pregnancies, ma! We have tried all means, but many still stop schooling and resort to staying at home. All they think of is marriage or getting pregnant. They have tried too many ways, right (there) at school, but children do what? Children still get pregnant*.

Often girls were presented as the ones choosing marriage and childbearing over schooling. But, in other discussions parents were said to place pressure on their children to give birth in order to secure their future, in the same vein alleviating their households of a mouth to feed: *They are also having pressure at home, ‘Ahh your friends at your age are already married. What are you doing?’ You see, so they want to get them married* (Coordinator, Health Centre). The economic dimension inherent in customary institutions like the bride-wealth, the exchange element in sexual relations and the high value placed on fertility and marriage hence emerged both subtly and through more direct statements.

### About peers and parents: patterns of transformation and continuity

The rapid transformation of culturally embedded norms and values was argued to lie beneath the rapidly shifting social scenario characterized by more premarital childbearing and school dropout. Informants complained about an increasing independence from parents: *There is nothing guarding the young ones, there is no-one to protect them…there is nothing guarding them really* (Ward Councilor). Youth were said to increasingly behave like ‘*mpengele’*, a type of bean that does not soften: *you cook it and cook it, but it doesn’t get cooked* (Ward Councilor). The implication was expressed as a loss of control over youth’s conduct: *Today they don’t care, they become pregnant, they get married. They do what? They think it doesn’t matter* (Nurse, Health Centre). Peers were said to play an increasingly important part as role models and to replace parental authority: *Because this one is doing it, I also need to do it. Because the one there is having a biscuit, while I’m not having any, I need to get one also. When they ask ‘where did you get the vanilla biscuit?’ the answer is ‘you should have a boyfriend.’ You see, it’s the peer pressure’* (Nurse, Health Centre). Knowledge about reproductive and sexual health was moreover increasingly said to be transmitted through peers, and was commonly said to be based on myths rather than knowledge in a context where health education in school hardly touched upon sexuality and on how to prevent pregnancy.

#### ‘*You cannot just eat my money’*

While the relations of socialization were said to be transforming at a rapid speed, the gendered norms seemed to be characterized by a substantial degree of continuity. The subordination of girls seemed to be sustained partly through unequal access to assets and cash placing girls in poor negotiating positions making them vulnerable. Girls were for example presented as having very poor ability to negotiate the use of the contraception. A Community Health Volunteer shared her experience of girls’ vulnerability saying: *Boys refuse to use condoms, they just go in direct.* Girls similarly referred to their talks with their boyfriends where they had little influence, partly related to their weak position when ‘indebted’ due to gift giving: *Men refuse to use a condom because they say that ‘you cannot just eat my money, then start putting in your ideas of a condom* (FGD girls).

A comprehensive reproductive health curriculum was in the process of being implemented in Zambian schools at the time of our study. However, in practice the teaching of sexuality and reproduction was said to continue to be both sporadic and superficial, a state mirrored in the lack of knowledge and misconceptions on sexual and reproductive health issues encountered during the interviews. Access to contraception, even at health centers, was moreover said to be challenging making it difficult for sexually active youth to avoid pregnancy.

Many of our adult informants supported a strict approach to reproductive and sexual health education in schools and access to contraception, preaching sexual abstinence prior to marriage as the only viable approach. However, others were more ambivalent, and particularly health workers and girl informants found the lack of availability of contraception and youth-friendly services problematic. A Nurse carefully stated: *Maybe if we just go back to the policy makers, because we know that nowadays people are getting pregnant as early as 14/15, so if they can allow family planning issued even in schools* (Maternal and Child Health Coordinator). A girl observed: *The only thing is, like for those who are not yet pregnant, if they could start taking them for family planning so that they continue their school.* Another communicated the point even more clearly: *Girls should be going for injections so that they complete school* (girl).

## Discussion

Drawing upon a social constructivist lens we identified simultaneous and seeming opposing lines of reasoning in our material; the officially endorsed discourse on early pregnancy as a problem and the schooling of girls as a solution met a parallel discourse that emphasized early childbearing as social and economic security. There was a constant juggling between these two major discourses that emerged side by side. The latter discourse sprang out of a local context where material scarcity was stated to motivate parents to marry off their daughters for bride-wealth, and where the need for cash and local gender - power dynamics favored early pregnancy. The high culturally constituted value placed on fertility and motherhood added to the complexity. These findings are largely in line with and complement the existing body of literature on adolescent pregnancy and early childbearing from Zambia and its neighboring countries [13, 20, 22]. In the following section we will particularly emphasize the importance of paying attention to such parallel discourses, and indicate their importance for intervention research. We argue that both discourses are concerned with securing the social and economic future of girls and their families, while the means to attain this goal are very differently defined.

### Parallel discourses on adolescent pregnancy in rural Zambian communities

As seen above, the official discourse was voiced loud and clear during the interviews. Informants in every category would praise schooling opportunities for girls and the delay of pregnancy and marriage as a central developmental strategy. Indeed, the most readily accessible discourse encountered among our informants was in line with the official stance where the problematic health-, educational and poverty related implications of early childbearing were emphasized. Delays of pregnancy and enhanced education for girls thus seemed to enjoy general support as part of the national strategy on poverty alleviation and development of Zambian rural communities. This dominant discourse was in the present context commonly underpinned and strengthened by Christian moral norms that view premarital sexual activity as inappropriate and immoral. A strongly advocated argument was that the dynamics behind the increase in early pregnancy rates were linked to the disintegration of culturally and religiously sanctioned normative patterns opening up for adolescents’ indulgence in sexual relations. Early pregnancy was thus in this context synonymous with pregnancy out of wedlock, as adolescent pregnancy within the frames of marriage was not a topic raised.

The dominant and publicly endorsed discourse presenting teenage pregnancy as a problem and schooling as a solution operated side by side with a clear, albeit not as loud informal discourse, that communicated alternative views and understandings of early childbearing. We saw that the parallel discourse commonly took the form of commentaries on how ‘parents” would entice their children to marry - and receive the bridewealth - rather than go on with their schooling, or on the manner in which ‘girls’ wished to marry and get pregnant rather than go to school. As demonstrated above, our informants would not readily present such alternative views with reference to themselves, and would commonly not hold these views or positions as their own. Rather, informants would bring up thoughts and practices that appeared to be at odds with the official stand with reference to ‘other’ community dwellers. They would point to others’ outdated views, positions, priorities and conduct - for example by referring to parents’ love for lobola and cattle or girls’ love for material things, boyfriends, marriage and pregnancy - rather than for education. Such positions were commented upon as out of touch with modern development thinking, and as failing to contribute to the aims of the government’s fight against early childbearing and school dropout in Zambian communities.

In a similar manner Jewkes et.al [22]. report from a South African setting how the political pressure to delay marriage, pregnancy and childbearing relates to locally grounded rationality and understanding of fertility and motherhood (p.734). They describe how in a South African context early pregnancy may be experienced as a rational option for young girls. Macleod [10] and Mkhwanazi [27] have demonstrated how community dwellers may find a number of positive dimensions in early pregnancy which are silenced or at least not fully acknowledged in the public discourse. Both the South African and Zambian official discourses commonly voice contrasting perceptions on early pregnancy taking place within and outside the frames of marriage, praising the former and condemning the latter. Although most women hope to bear children within the frames of marriage, there are many reasons why early pregnancy may be perceived as a rational option despite overt disapproval, a point made by authors from different parts of the world [41–44]. A child may not necessarily impair a girls’ future; for many adolescents and parents premarital early pregnancy will demonstrate female fertility, the father of a child may marry the girl and thus potentially secure her future etc. Hence, also a premarital pregnancy may be experienced as social and economic security for a girl.

Social constructionist approaches underline the inherent links between knowledge and social processes and the link between knowledge and social action [38]*.* That is, socially constructed discourse has social implications, meaning that the two contrasting discourses laid out above have their counterparts or place in social life. This point is of substantial relevance for interventions and intervention research, as it cautions us against basing our conclusions on - in this case – readily communicated support to a publicly endorsed policy agenda, and opens up to alternative local reasoning. Acknowledging the dynamics between the different discourses encountered locally moreover enhances our understanding of how both discourses influence adolescent sexual practices. This in turn facilitates an understanding of the continued high prevalence of early pregnancy within a context of substantial political mobilization against early childbearing and early marriage.

### Early pregnancy and social and economic security

Both the official and the alternative discourse address concerns with securing or improving the livelihood and futures of girls. The official discourse frames delayed age of first pregnancy with reference to young women’s health and to girls’ knowledge, empowerment and sustanance. The parallel discourse being more accepting of early childbearing similarly reveals concerns with sustenance, that is, securing the social and economic future of the girl. The economic dimension of early childbearing surfaced through statements about poor parents arranging early marriages for their daughters due to a failing to pay schoolfees or in order to receive the bridewealth or parents encouraging their daughters to enter relations with boyfriends ‘to get something to eat’. Resource constraints also emerged as an inherent part of the exchange-element of sexual relations and of early childbearing itself, as a baby could lead to marriage and thus potentially secure a girl’s future.

Pradhan et al. ‘s systematic review [12] on factors associated with pregnancy among adolescents in low-income and lower-middle income countries, conclude that structurally embedded factors, such as socio-economic position, limited education and insufficient access to and use of contraception are consistently found to imply ‘risks for pregnancy among adolescents’ (p. 1). The authors conclude that whether in resource-constrained or in well-resourced contexts, low socioeconomic position appears to increase the risk of pregnancy among adolescents. They write that there are additional risks specific to resource constrained settings, and point to early marriage, the dowry institution and inaccurate beliefs about contraception. While we support Pradham et al’s main reasoning, we argue that also in the analysis of factors such as early marriage, the bride wealth institution, inaccurate beliefs about contraception and the hailing of fertility, economic- or structural aspects surface. Resource constraints have long since proven to be an integral part of culturally constituted ways of handling pregnancy- and childbearing [42, 45].

Greenhalgh [16] has in classical ethnographic studies revealed the importance of taking seriously the political economy of fertility if we are to grasp the forces underlying the timing of childbearing. She emphasizes that in attempts to enhance our understanding of apparent irrational early childbearing, careful examination is needed of how dynamics of early motherhood is often deeply embedded in relationships of power and economic disadvantage (p. 15).

While pointing to the poverty related dimension of early pregnancy is surely not new, the study findings bring to the forefront of attention the importance of the structural factors and economic constraints that condition and reinforce a culture of early childbearing. Hence, we argue that structural dynamics must be addressed and appropriated as a key part of any intervention or policy attempt to confront the issue of early pregnancy. Indeed, lest the harshly experienced resource constraints in rural Zambia are made an inherent part of an intervention to reduce early pregnancy, there is little reason to believe that permanent transformations of patterns of early pregnancy will take place.

### Strengths and limitations

Although substantial focus was placed on the transcription and translation process, research material in need of language translation is confronted with inherent challenges in terms of potential loss of meaning. Only two of the co-authors took part in the data collection. All co-authors do however have extensive experience from long term reproductive health research in diverse African settings, and are thus familiar with debates surrounding early pregnancy, schooling and public policy from similar contexts. The research was carried out within a context of a strong public policy agenda against early pregnancy, and due to the challenge of voicing alternatives to publicly endorsed views our study could have benefitted from an ethnographic component allowing for longer term exploration of fluctuations in discourse and of the articulation between talk and practice. While the study findings are context specific and situated in a rural Zambian context, it is likely that findings and conclusions have relevance also for other rural Zambian settings. The material may moreover have some relevance also in other rural sub-Saharan contexts where poverty is rampant and early pregnancy rates are high.

## Conclusions

Informants in the study strongly supported the present global health- and educational policies that denounce adolescent pregnancy and strongly encourage extended schooling for girls. The study simultaneously points to a parallel discourse revealing the value attached to early pregnancy among the informants. The latter and alternative discourse emerged as fundamentally embedded in poverty- and economic and social security-related concerns. We argue that if the agenda against early pregnancy as a threat to girls’ health, empowerment and schooling is to gain terrain in rural Zambian communities, it needs to address the resource constraints that condition cultures of early childbearing.

## Supplementary information

**Additional file 1.** Interview guide ‘Girls in and out of school’.

**Additional file 2.** Interview guide ‘Girls not mothers in and out of school’.

**Additional file 3.** Interview guide: ‘Girls with babies in and out of school’.

**Additional file 4.** Interview guide ‘Teachers, health workers, community leaders’.

**Additional file 5.** Interview guide ‘District officials’.

**Additional file 6.** Interview guide ‘Parents’.

**Additional file 7.** Topic guide ‘Girls and boys’ (separate groups).

**Additional file 8.** Topic guide ‘Adults’.

## Data Availability

The data material can be made available on request through the last author.

## Abbreviations

DHS: Demographic and Health Survey
FGD: Focus Group Discussion
RISE: Research Initiative to Support the Empowerment of Girls
SRH: Sexual and Reproductive Health
