# Supplementary material for: Vanilla bisquits and lobola bridewealth: parallel discourses on early pregnancy and schooling in rural Zambia
Source: BMC Public Health. 2020 Oct 1;20:1485. doi: 10.1186/s12889-020-09555-y (PMC7528241; doi:10.1186/s12889-020-09555-y)
Supplement: Supplementary file 1 — Additional file 1. Interview guide ‘Girls in and out of school’. [file 12889_2020_9555_MOESM1_ESM.docx]

**INTERVIEW GUIDE GIRLS - IN AND OUT OF SCHOOL (NOT MOTHERS)**

**Remember to probe, get concrete examples and spend time (up to 90 minutes). Let the informant speak at length and make sure that you use this guide only as a guide in the interview process and not as a list of questions to be covered one after the other.**

**Potential probes = P**

**A Introduction**

Can you please tell me a little bit about yourself and your family?

P: How old are you? Did you grow up in this community, how many brothers and sisters, how many went to school? Are you in school now? Who do you currently live with? What do your parents/guardians do for a living?

**B Marriage**

Can you tell me about marriage customs in this community?

P: How do marriages commonly occur? How are marital partners commonly chosen? What role should parents play over the marriage of their children? (boys vs girls)

At what point are men and women / girls and boys expected to get married?

P: approximate age, biosocial signs, economy, schooling, pregnancy, other?

What would in your opinion be an ideal marriage in this community? Please give examples

Would you like to share your thoughts about what would be a non-desirable marriage? Please give examples

Can you please reflect on the relationship between schooling and marriage?

P: What are your thoughts about girls who get married while in schools? Do you have any experience with girls in your family getting married while in school? How common / acceptable is marriage among primary school girls? Do you see any situation where education becomes a barrier to marriage, for instance staying too long in school.

**C Pregnancy and childbirth**

How important is it to have children for a man versus a woman in this community?

When and under which circumstances is it expected and desired that a woman gives birth here?

P: age, physical maturity, marriage, economic security, families agree, love, other? Examples

When is pregnancy unacceptable or unwanted in this community?

P: for age, immaturity, schooling, poverty, outside of marriage, other? Examples

How common and acceptable is pregnancy among unmarried girls in the community?

P: Examples. Community reactions

How common are pregnancies among primary school going girls in the community?

P: Examples. Community reactions

How common are pregnancies among secondary school going girls in the community?

P: Examples. Community reactions

What in your view are the main reasons why girls in this community become pregnant?

P: Desire to become a mother, social pressure, lack of knowledge of reproductive health and rights, lack of access to contraception, lack of negotiating power, rape, relations to older men for economic reasons, other?

What are your personal thoughts / opinions about girls who get pregnant while in school?

P: How do you think it will affect their lives?

Do you have grandchildren?

P: Do you have any experience of unwanted pregnancy in your family? Were any of your grandchildren born while your children were still in school? Please tell

**D Education and learning environment**

**For girls in school:**

- Can you describe what you like about school?
- P: subjects, friends, teacher.
- Are there things that you do not like about school? If yes, can you describe what you do not like? (school road, harsh teachers, absent teachers, relations to other pupils, sanitation, facilities etc)
- Are you able to attend school every day? If not what hinders you?
- P: work at home, care for siblings, sick relatives, distance, poverty, lack of food, lack of motivation and interest, menstruation, poor toilet facilities
- Do you feel that your teachers treat boys and girls equally at school? If no, can you please give an example?
- Do you feel that you are able to participate on equal terms as boys in the classroom?

**For girls not in school:**

- What caused you to drop out school?
- P: work at home, care for siblings, sick relatives, distance, poverty, lack of food, lack of motivation and interest, menstruation, poor toilet facilities
- Are there things that you do not like about school? If yes can you describe what you do not like? (school road, harsh teachers, absent teachers, relations to other pupils, sanitation, facilities etc)
- Do you feel that your teachers treat boys and girls equally at school? If no, can you please give an example?
- Do you feel that you were able to participate on equal terms as boys in the classroom?

**III: The value of education**

- Do you think school is important in preparing someone for the future ahead?
- Do you think education is important for your future life?
  - If yes, what is the most important aspect of going to school? (life skills, strengthen independence and autonomy, protecting health, job opportunities etc)
  - If no, why not? (not relevant for life ahead, no income, difficult to get paid work, prevents me from finding a husband/marrying/having children).
  - What do you think is the ideal number of years in school? Is there a difference between girls and boys?
- How do your peers value education?
  - Is there any difference in how boys and girls value education?
- How do parents in this area value education?
  - Do parents in this area send all their children to school?
  - Is there any difference in how they value the importance for boys and girls?
  - What do parents perceive as the ideal number of years in school for their children?

How many years have you been in school?

Can you describe what you like about school?

How do you think the school system prepares youth for life ahead?

P: Potential differences girls / boys

How important do you think education is for a good life?

What do you think is the ideal number of years in school?

P: differences between girls and boys?

In your opinion is school drop-out a big problem in this community?

P: Boys vs girls? What would be the main reasons for school drop out among girls? (poverty, need for labour at home, food shortage at school, distance, security, pregnancy, other?)

How important do you think pregnancy is for school drop-out?

What happens when a girl becomes pregnant while in school? Please give examples.

**E Interventions**

What do you think should be done to help girls achieve their educational goals while at the same time meeting community expectations (marriage and childbearing?)

What do you think is the best way to encourage girls to stay in school/prevent drop outs?

a) Economic support (P: stipends, school uniforms, school meals, transport etc)

b) Improved learning environment (P: improved reproductive and sexual health education inside school/school clubs, from health personnel, at community level, sanitation, improved access to contraception, school meals, other)

c) Improve security on school road.

**I: Introduction**

- Smalltalk
- How old are you?
- Who do you live with?
- Are your guardians/parents farmers/herders/in business?
- Did any of your guardians/parents go to school?
- Do you have sisters and brothers? How old are they? Are they going to school?
- Are your guardians/parents supportive of education for their children?

**II: Learning environment:**

- What caused you to drop out school? (work at home, care for siblings, sick relatives, distance, poverty, lack of food, lack of motivation and interest, menstruation, poor toilet facilities)
- Are there things that you do not like about school? If yes can you describe what you do not like? (school road, harsh teachers, absent teachers, relations to other pupils, sanitation, facilities etc)
- Do you feel that your teachers treat boys and girls equally at school? If no, can you please give an example?
- Do you feel that you were able to participate on equal terms as boys in the classroom?

**III: The value of education**

- Do you think school is important in preparing someone for the future ahead?
- Do you think education is important for your future life?
  - If yes, what is the most important aspect of going to school? (life skills, strengthen independence and autonomy, protecting health, job opportunities etc)
  - If no, why not? (not relevant for life ahead, no income, difficult to get paid work, prevents me from finding a husband/marrying/having children).
  - What do you think is the ideal number of years in school? Is there a difference between girls and boys?
- How do your peers value education?
  - Is there any difference in how boys and girls value education?
- How do parents in this area value education?
  - Do parents in this area send all their children to school?
  - Is there any difference in how they value the importance for boys and girls?
  - What do parents perceive as the ideal number of years in school for their children?

**IV: The challenge of school dropout:**

- How big is the problem of school drop outs in this school? Are there any differences between girls and boys?

Potential probes:

- - How important do you think poverty is for school drop-out? Are they related to for instance lack of funds for uniforms and contributions (fees) to the school; labour at home, lack of food
  - How important do you think distance to school is for school drop-out
  - How important do you think insecurity on the school road is for school drop-out (elopement, rape)
- How important do you think pregnancy is for school drop-out?

Potential probes:

- - Do you know anybody who has dropped out due to pregnancy?
  - What happens to a girl who becomes pregnant while in school?
  - Is it possible for a pregnant girl to stay in school?
  - How would co-pupils and teachers perceive having a pregnant girl in class?
  - Is it possible/common to return to school after giving birth?
  - How would she be welcomed and fit in the class upon return?
- What are the reasons why schoolgirls become pregnant (lack of knowledge of reproductive health and rights, lack of negotiating power, rape, relations to older men, accidental, preference etc)

**V: Dreams and hopes for the future**

- What is most important for you to achieve in life? (becoming a mother, having a husband, getting my own home, having an income to sustain my children, getting education, being knowledge and independent, getting a job, being able to take care of siblings and old relatives/parents). What are the characteristics that you would treasure in a husband? (good job, kindness, politeness, respect, fertility, education?)
- If you imagine your life 5 years from now: How do you hope/think it will look like?
- Is there a girl in this neighbourhood that you are looking up to in terms of life goals?

**VI: Retaining girls in school**

- Is drop-out /push out of girls a particular problem in your school? At what
- Is it important to encourage more education for girls? If yes, what do you think is the best way to encourage girls to stay in school/prevent drop outs and ?
- Potential probes:
  - Economic support (stipends, school uniforms, school meals, transport etc)
  - Improved learning environment (sanitation, strengthening school clubs for reproductive health, more relevant content, school meals)
  - Improve security on school road (companionship, transport)
  - Others?
