# Supplementary material for: Vanilla bisquits and lobola bridewealth: parallel discourses on early pregnancy and schooling in rural Zambia
Source: BMC Public Health. 2020 Oct 1;20:1485. doi: 10.1186/s12889-020-09555-y (PMC7528241; doi:10.1186/s12889-020-09555-y)
Supplement: Supplementary file 2 — Additional file 2. Interview guide ‘Girls not mothers in and out of school’. [file 12889_2020_9555_MOESM2_ESM.docx]

**INTERVIEW GUIDE GIRLS OUT OF SCHOOL (non-mothers)**

**Remember to probe, get concrete examples and spend time (up to 90 minutes). Let the informant speak at length and make sure that you use this guide only as a guide in the interview process and not as a list of questions to be covered one after the other.**

**Potential probes = P**

**A: Introduction**

- Can you please tell me a bit about yourself and your family?

P: How old are you? Did you grow up in this community, how many brothers and sisters? Do you have a partner? Who do you currently live with? What do your guardians /husband/partner do for a living? What do you do for a living?

- How do you experience your life in the village? Do you have many peers in the same situation (not yet mothers and not going to school)
- What are main daily chores?

**B: Pregnancy and childbirth**

- Have any of your friends become mothers?
  - If yes, how is this perceived by her peers?
- Among your peers, when and under which circumstances is it expected and desired that a woman/girl gives birth?
  - P: age, physical maturity, marriage, economic security, families agree, love, other? Examples
- How common and acceptable is pregnancy among unmarried girls in this community? To what extent is it age related?
  - P: Examples. Community and peer reactions.
- When and under which circumstances is pregnancy unacceptable or unwanted in this community?
  - P: age, immaturity, schooling, economic insecurity, outside of marriage, other? Examples
- What do you think are the reasons why young girls become pregnant?
  - P: Desire to become a mother, social pressure, lack of knowledge of reproductive health and rights, lack of access to contraception, lack of negotiating power, rape, relations to older men for economic reasons, accidental

**C: Schooling and the value of education**

- Did you ever attend school? If yes, for how many years?
  - P: What caused you to drop out school? (work at home, care for siblings, sick relatives, distance, unsafe school road, poverty, harsh teachers, lack of food, lack of motivation and interest, menstruation, poor toilet facilities)
  - P: Would you have liked to continue school? What kind of opportunities do you think that would have given you?
- In this community, who are the girls that you look up to?
  - P: Girls going to school, not going to school, mothers/non-mothers . Concrete examples
- Do you think education is important for your future life?
  - P: If yes, what is the most important aspect of going to school? (life skills, strengthen independence and autonomy, protecting health, job opportunities etc)
  - P: If no, why not? (not relevant for life ahead, no income, difficult to get paid work, prevents me from finding a husband/marrying/having children).
  - P: What do you think is the ideal number of years in school? Is there a difference between girls and boys?
- How do your peers value education?
  - Is there any difference in how boys and girls value education?
- Do you have many friends who do not go to school? Why did they discontinue / never start schooling:
  - P: poverty, lack of funds for uniforms and contributions (fees) to the school; labour at home, lack of food
  - P: Long distance to school
  - P: Insecurity on the school road (elopement, rape)
- Do you know anybody who stopped school due to pregnancy?
  - P: If yes, what happened to her? Example

**D: Dreams and hopes for the future**

- What is most important for you to achieve in life?
  - P: becoming a mother, having a husband, getting my own home, having an income to sustain my children, getting education, being knowledge and independent, getting a job, being able to take care of siblings and old relatives/parents).
- If you imagine your life 5 years from now: How do you hope/think it will look like?
- Is there a girl in this neighbourhood that you are looking up to in terms of life goals?

**E: Interventions**

- What kind of help do you think should be put in place to prevent unwanted pregnancies for girls in this community?
- What do you think should be done to help girls achieve their educational goals while at the same time meeting community expectations (marriage and childbearing?)
- What do you think would be the best way to support girls who want to stay in school?
  - P: Economic support (stipends, school uniforms, school meals, transport etc)
  - P: Improved learning environment (improved reproductive and sexual health education inside school/school clubs, enhanced sanitation at school, information from health personnel at school /outside school, campaigns at community level, improved access to contraception, school meals, other)
  - P: Improve security on school road, construct more schools to reduce distance? Other?
