# Supplementary material for: Vanilla bisquits and lobola bridewealth: parallel discourses on early pregnancy and schooling in rural Zambia
Source: BMC Public Health. 2020 Oct 1;20:1485. doi: 10.1186/s12889-020-09555-y (PMC7528241; doi:10.1186/s12889-020-09555-y)
Supplement: Supplementary file 3 — Additional file 3. Interview guide: ‘Girls with babies in and out of school’. [file 12889_2020_9555_MOESM3_ESM.docx]

**INTERVIEW GUIDE FOR GIRLS UNDER 18 WITH A HISTORY OF EARLY PREGNANCY**

**Remember to probe, get concrete examples and spend time (up to 90 minutes). Let the informant speak at length and make sure that you use this only as a true guide in the interview process, and not as a list of questions to be covered one after the other.**

**Potential probes = P**

**A Introduction**

Can you please tell me a bit about yourself and your family?

P: How old are you? Did you grow up in this community, how many brothers and sisters, how many went to school? How many children do you have? Marital status? Are you in school now? Who do you currently life with? What do your guardians /husband/partner do for a living?

**B Experience with early motherhood**

-What does being a mother mean to you?

P: the desire to have a child, the value of motherhood, love for the baby.

-What kind of support do you get to care for your child?

P: From whom do you get support? parents, grandparents, siblings, partner/spouse, teachers, other community members?

-What challenges are you facing in taking care of your child (ren)?

P: lack of support, financial difficulties, school related challenges, stigma, other?

**C Experience with early pregnancy**

-Can you please tell me about the circumstances behind the pregnancy?

P: Was the pregnancy planned / unplanned? How old is the father of the child? Are you together with the father of the child now? At what age did you first get pregnant? Were you in school when you got pregnant?

-What came to your mind when you learnt that you were pregnant?

P: happiness, fear, panic?

-How did you react when you understood that you were pregnant?

-Who did you disclose your pregnancy to?

P: How was the reaction of your partner/husband / parents / guardians / friends / community / teachers?

-At what point in your pregnancy did you stop schooling? Was your condition disclosed

while in school?

-What were the reasons for discontinuing school?

P: Pressure from school, community, church? Do you have plans to re-enter school later? Who guided you in terms of choices of schooling when you were pregnant? Were you in agreement with the decisions reached and the guidance provided to you?

**In school girls:**

- How did you experience becoming pregnant while in school?

- Was your pregnancy disclosed while in school?

- How did your people react to your pregnancy?

P: your own peers’, parents’, teachers’, boy friend

- What decisions were made about your schooling?

P: Who were involved in the decision making?

- Were you in agreement with the decisions reached and the guidance provided to you?
- Did you discontinue school for some time before re-entering?

P: If yes: For how long were you absent from school?

- How did you experience re-entering school?

**D Plans and dreams for life ahead**

-What are your dreams for your future life? (motherhood, education, a job, a good husband etc)

P: Can you please tell me about a girl in this community who has been able to fulfil what you dream of?

-Is there anything that you would have liked to do differently if you got another chance? If

yes, what would that be?

-If you wish to postpone another pregnancy, would you know what to do or where to go to seek advice and services?

P: knowledge, access to contraception

-Would you consider going back to school if given a chance?

-What advice would you give to other teenagers about sexual and reproductive health?

- In this community, who are the girls that you look up to?

P: with schooling/no schooling, mothers/non-mothers . Concrete examples

**E Interventions**

-What kind of help do you think should be put in place to prevent unwanted pregnancies for girls in this community?

-What do you think should be done to help girls achieve their educational goals while at the same time meeting community expectations (marriage and childbearing?)

-What do you think is the best way to encourage girls to stay in school/prevent drop outs?

a) Economic support (P: stipends, school uniforms, school meals, transport etc)

b) Improved learning environment (P: improved reproductive and sexual health education inside school/school clubs, enhanced sanitation at school, information from health personnel at school /outside school, campaigns at community level, improved access to contraception, school meals, other)

c) Improve security on school road, construct more schools to reduce distance? Other?
