# Supplementary material for: Vanilla bisquits and lobola bridewealth: parallel discourses on early pregnancy and schooling in rural Zambia
Source: BMC Public Health. 2020 Oct 1;20:1485. doi: 10.1186/s12889-020-09555-y (PMC7528241; doi:10.1186/s12889-020-09555-y)
Supplement: Supplementary file 4 — Additional file 4. Interview guide ‘Teachers, health workers, community leaders’. [file 12889_2020_9555_MOESM4_ESM.docx]

**INTERVIEW GUIDE WITH HEALTH WORKERS, TEACHERS AND COMMUNITY LEADERS**

**Remember to probe, get concrete examples and spend time (up to 90 minutes). Let the informant speak at length and make sure that you use this only as a true guide in the interview process, and not as a list of questions to be covered one after the other.**

**Potential probes = P**

**A Introduction (small talk)**

What is your role/work in this community? How long have you worked and lived here? Is this your home area? Where else have you lived / worked?

**B Marriage**

At what point are men and women / girls and boys expected to get married in this community?

P: approximate age, biosocial signs, economy, schooling, pregnancy, other? What is perceived to be an early marriage in this community?

How important is it to be married before you start childbearing in this community?

Can you please reflect on the relationship between schooling and marriage?

P: How common is marriage among primary school girls? How common / acceptable is marriage among secondary school girls?

What are your thoughts about girls who get married while in school?

P: May marriage get in the way of schooling? Do you see any situation where education becomes a barrier to marriage/childbearing, e.g. staying long in school?

What do you think are the main reasons for early marriages in this community?

P: What role do you think parents/guardians and community cultural practices play in ‘early’ marriages in this community?

What efforts are available to curb ‘early’ marriages in the community? Potential efforts planned in the near future?

P: What are the main challenges in implementing efforts to curb ‘early’ marriage? What do you think are/would be the best strategies to reduce early marriage in this community?

**C Pregnancy and childbirth**

When and under which circumstances is it expected and desired that a woman gives birth?

P: age, physical maturity, marriage, economic security, families agree, love, other? Examples

When is pregnancy unacceptable or unwanted in this community?

P: age, immaturity, schooling, economic insecurity, outside of marriage, other? Examples

How common and acceptable is pregnancy among unmarried girls in this community?

P: Examples. Community reactions

How common are pregnancies among primary school going girls in this community?

P: Examples. Community reactions

How common are pregnancies among secondary school going girls in this community?

P: Examples. Community reactions

Have you seen any changed in the last five years in the number of girls who become

pregnant before age 16?

What are your opinions about girls who get pregnant while in school?

P: How do you think it will affect their lives?

What are the main reasons why girls become pregnant in this community?

P: Desire to become a mother, social pressure, lack of knowledge of reproductive health and rights, lack of access to contraception, lack of negotiating power, rape, relations to older men for economic reasons, other?

What have been your work related experiences with issues relating to early pregnancy

among girls?

P: Ask for concrete examples

What are the common reactions to pregnancy among school girls?

P: shame, marriage, discontinuation of schooling, can a girl who becomes married continue to stay in school, can a girl re-enter school after giving birth?

**D Education**

How do people in this community value education for their daughters / sons?

P: How long do boys and girls commonly go to school in this community?

How do you think the school system prepares youth for life ahead?

P: How do you think secondary school changes the possibilities for girls in terms of employment, marriage, life skills, independence/autonomy and social participation? Potential differences girls / boys?

Do you think it may be difficult for parents to send all their children to school?

P: Reasons: economy, need for labour, fear of pregnancy, unsafe school road, other?

Do you see any opportunities that children and youth may miss because of being

enrolled in school?

P: boys vs girls?

In your opinion is school drop-out a big problem in this community?

P: Boys vs girls? At what level? What are the main reasons for school drop-out among girls? (poverty, need for labour at home, food shortage at school, distance, security, lack of role models, difficult to get enrolled/lack of places, lack of interest/limited social support, pregnancy, other?)

How important do you think pregnancy is for school drop-out?

What happens when a girl becomes pregnant while in school? Please give examples.

How is the policy of girls re-entering school after childbirth working in this community?

P: Have you come across girls who have finished school after childbirth?

***Teachers****:*

Do the pupils get sexual and reproductive health education at this school?

Do you teach sexual and reproductive health education in any of your classes?

P: What do you focus on? Do you feel comfortable teaching about these topics?

How often is the school visited by health workers?

What reproductive and sexual health services do health workers provide when they visit the school?

How do the pupils experience the visits of the health workers? How do you view these visits?

***Health workers:***

How often do you visit schools in the district as part of your work?

What services do you offer and what information do you provide when you visit schools?

Do the pupils get sexual and reproductive health education at this school?

Do you teach sexual and reproductive health education in any of your classes?

P: What do you focus on? Do you feel comfortable teaching about these topics?

***Community leaders:***

How do you consider the role of teachers and health workers in providing sexual and reproductive health education?

How do you consider the role of the church in providing sexual and reproductive health education?

How do you consider the role of traditional institutions in providing sexual and reproductive health education?

**E Interventions**

What efforts or programs have been put in place to curb unwanted pregnancy?

P: Are there any challenges in implementing efforts to curb teenage pregnancy?

What efforts or programs have been put in place to prevent girls from dropping out of school due to pregnancy?

What do you think should be done to help girls achieve their educational goals while at the same time meeting community expectations (marriage and childbearing?)

What do you think is the best way to encourage girls to stay in school/prevent drop outs?

a) Economic support (P: stipends, school uniforms, school meals, transport etc)

b) Improved learning environment (P: improved reproductive and sexual health education inside school/school clubs, enhanced sanitation at school, information from health personnel at school /outside school, campaigns at community level, improved access to contraception, school meals, other)

c) Improve security on school road, construct more schools to reduce distance? Other?
