# Supplementary material for: Vanilla bisquits and lobola bridewealth: parallel discourses on early pregnancy and schooling in rural Zambia
Source: BMC Public Health. 2020 Oct 1;20:1485. doi: 10.1186/s12889-020-09555-y (PMC7528241; doi:10.1186/s12889-020-09555-y)
Supplement: Supplementary file 5 — Additional file 5. Interview guide ‘District officials’. [file 12889_2020_9555_MOESM5_ESM.docx]

**KEY INFORMANT INTERVIEW GUIDE FOR STAFF AT DISTRICT LEVEL**

District Community Health Office: Maternal and child health officer

District Chiefs and Traditional Affairs office

District Education office

**Remember to probe, get concrete examples and spend time (up to 90 minutes). Let the informant speak at length and make sure that you use this only as a true guide in the interview process, and not as a list of questions to be covered one after the other.**

**Potential probes = P**

**A Introduction**

What is your role in the district administration? How long have you been working and living here? Is this your area of origin? Where else have you lived/worked?

**B Marriage**

At what point are men and women / girls and boys expected to get married in this district?

P: approximate age, biosocial signs, economy, schooling, pregnancy, other? What is perceived to be an early marriage in this district?

How important is marriage for childbearing in this district?

Can you please reflect on the relationship between schooling and marriage?

P: How common and acceptable is marriage among primary school girls? How common / acceptable is marriage among secondary school girls?

What are your thoughts about girls who get married while in schools?

P Can marriage get in the way for schooling? Do you see any situation where schooling / education becomes a barrier to marriage/childbearing?

What do you think are the main reasons for early marriages in this district?

P: What role do you think parents/guardians and community cultural practices play in ‘early’ marriages in this district?

*NB! What experiences do you have with issues of early marriage in your work?*

**C Pregnancy and childbirth**

When and under which circumstances is it expected and desired that a woman gives birth in this district?

P: age, physical maturity, marriage, economic security, families agree, love, other?

When is pregnancy unacceptable or unwanted in this district?

P: age, immaturity, schooling, economic insecurity, outside of marriage, other?

What are the common reactions to pregnancy among school girls?

P: shame, marriage, discontinuation of schooling, can a girl who becomes married continue to stay in school, can a girl re-enter school after giving birth?

How common and acceptable is pregnancy among unmarried girls in this district?

How common is pregnancy among girls in primary school in this district?

How common is pregnancy among girls in secondary school in this district?

Have you seen any change in the last five years in the number of girls who become

pregnant before age 16 in this area?

What are your opinions about girls who get pregnant while in school?

P: How do you think it will affect their lives?

What are the main reasons why girls become pregnant in this district?

P: Desire to become a mother, social pressure, lack of knowledge of reproductive health and rights, lack of access to contraception, lack of negotiating power, rape, relations to older men for economic reasons, other?

*NB! What is your work related experience with issues relating to early pregnancy among girls?*

**D Education**

How do people in this district value education for their daughters / sons?

P: How long do boys and girls commonly go to school in this district?

How do you think the school system prepares youth for life ahead?

P: How do you think secondary school changes the possibilities for girls in terms of employment, marriage, life skills, independence/autonomy and social participation? Potential differences girls / boys?

Do you think it may be difficult for parents to send all their children to school?

P: Reasons: economy, need for labour, fear of pregnancy, unsafe school road, other?

Do you see any opportunities that children and youth may miss because of being enrolled in school?

P: boys vs girls?

In your opinion is school drop-out a big problem in this district?

P: Boys vs girls? At what level? What are the main reasons for school drop-out among girls? (poverty, need for labour at home, food shortage at school, distance, security, lack of role models, difficult to get enrolled/lack of places, lack of interest/limited social support, pregnancy, other?)

How important do you think pregnancy is for school drop-out?

What happens when a girl becomes pregnant while in school? Please give examples.

How is the policy of girls re-entering school after childbirth working in this district?

P: Have you come across girls who have finished school after childbirth?

To what extent do the pupils get sexual and reproductive health education in school?

P: What is taught?

What reproductive and sexual health services do health workers provide for pupils?

**E Interventions**

What efforts or programs do you have in this district that to curb unwanted pregnancy?

P: probe for an overview of existing programs (district/regional/state, NGOs, WHOetc). What is your experience with these programs? What kinds of challenges are you confronted with in implementing efforts to curb unwanted pregnancy?

What efforts or programs have been put in place to prevent girls from dropping out of school due to pregnancy?

P: probe for an overview of existing programs (district/regiona/state, NGOs, WHOetc). What is your experience with these programs? What kinds of challenges are you confronted with in implementing efforts to curb school drop out related to unwanted pregnancy?

What efforts or programs do you have to prevent ‘early’ marriage? (probe to find out what some of these initiatives are and how they are working)

P: probe for an overview of existing programs (district/regiona/state, NGOs, WHOetc). What is your experience with these programs? What kinds of challenges are you confronted with in implementing efforts to curb school early marriage?

What do you think should be done to help girls achieve their educational goals while at the same time meeting community expectations (marriage and childbearing?)

What do you think is the best way to encourage girls to stay in school/prevent drop outs?

a) Economic support (P: stipends, school uniforms, school meals, transport etc)

b) Improved learning environment (P: improved reproductive and sexual health education inside school/school clubs, enhanced sanitation at school, information from health personnel at school /outside school, campaigns at community level, improved access to contraception, school meals, other)

c) Improve security on school road, construct more schools to reduce distance? Other?
