# Supplementary material for: Vanilla bisquits and lobola bridewealth: parallel discourses on early pregnancy and schooling in rural Zambia
Source: BMC Public Health. 2020 Oct 1;20:1485. doi: 10.1186/s12889-020-09555-y (PMC7528241; doi:10.1186/s12889-020-09555-y)
Supplement: Supplementary file 6 — Additional file 6. Interview guide ‘Parents’. [file 12889_2020_9555_MOESM6_ESM.docx]

**INTERVIEW GUIDE FOR PARENTS**

**Remember to probe, get concrete examples and spend time (up to 90 minutes). Let the informant speak at length and make sure that you use this guide only as a guide in the interview process and not as a list of questions to be covered one after the other.**

**Potential probes = P**

**A Family information**

Can you please tell me a about your family?

P: Who lives in this home? How many children do you have (boys/girls)? How do you earn a living?

**B Marriage**

Can you tell me about marriage customs in this community?

P: How do marriages commonly occur? How are marital partners commonly chosen? What role should parents play over the marriage of their children? (boys vs girls)

At what point are men and women / girls and boys expected to get married?

P: approximate age, biosocial signs, economy, schooling, pregnancy, other?

What would in your opinion be an ideal marriage in this community? Please give examples

Would you like to share your thoughts about what would be a non-desirable marriage? Please give examples

Can you please reflect on the relationship between schooling and marriage?

P: What are your thoughts about girls who get married while in schools? Do you have any experience with girls in your family getting married while in school? How common / acceptable is marriage among primary school girls? Do you see any situation where education becomes a barrier to marriage, for instance staying too long in school.

**C Pregnancy and childbirth**

How important is it to have children for a man versus a woman in this community?

When and under which circumstances is it expected and desired that a woman gives birth here?

P: age, physical maturity, marriage, economic security, families agree, love, other? Examples

When is pregnancy unacceptable or unwanted in this community?

P: for age, immaturity, schooling, poverty, outside of marriage, other? Examples

How common and acceptable is pregnancy among unmarried girls in the community?

P: Examples. Community reactions

How common are pregnancies among primary school going girls in the community?

P: Examples. Community reactions

How common are pregnancies among secondary school going girls in the community?

P: Examples. Community reactions

What in your view are the main reasons why girls in this community become pregnant?

P: Desire to become a mother, social pressure, lack of knowledge of reproductive health and rights, lack of access to contraception, lack of negotiating power, rape, relations to older men for economic reasons, other?

What are your personal thoughts / opinions about girls who get pregnant while in school?

P: How do you think it will affect their lives?

Do you have grandchildren?

P: Do you have any experience of unwanted pregnancy in your family? Were any of your grandchildren born while your children were still in school? Please tell

**D Education**

How do you think the school system prepares youth for life ahead?

P: Potential differences girls / boys

How important do you think education is for a good life?

What do you think is the ideal number of years in school?

P: differences between girls and boys?

Do any of your children go to school?

P: If yes, who of your children go to school? (boys/girls)

How did you choose whom to send to school/continue to secondary school? (gender, school performance, need for labour, economic situation, the child’s wish)

How do your children like being in school?

Has it at times been difficult for you to send your children / all children to school?

P: Reasons: economy, need for labour, fear of pregnancy, unsafe school road, other?

Do you see any opportunities that children and youth may miss because of being enrolled in school?

P: boys vs girls?

In your opinion is school drop-out a big problem in this community?

P: Boys vs girls? What would be the main reasons for school drop out among girls? (poverty, need for labour at home, food shortage at school, distance, security, pregnancy, other?)

How important do you think pregnancy is for school drop-out?

What happens when a girl becomes pregnant while in school? Please give examples.

**E Interventions**

What do you think should be done to help girls achieve their educational goals while at the same time meeting community expectations (marriage and childbearing?)

What do you think is the best way to encourage girls to stay in school/prevent drop outs?

a) Economic support (P: stipends, school uniforms, school meals, transport etc)

b) Improved learning environment (P: improved reproductive and sexual health education inside school/school clubs, from health personnel, at community level, sanitation, improved access to contraception, school meals, other)

c) Improve security on school road.
