# Supplementary material for: Vanilla bisquits and lobola bridewealth: parallel discourses on early pregnancy and schooling in rural Zambia
Source: BMC Public Health. 2020 Oct 1;20:1485. doi: 10.1186/s12889-020-09555-y (PMC7528241; doi:10.1186/s12889-020-09555-y)
Supplement: Supplementary file 7 — Additional file 7. Topic guide ‘Girls and boys’ (separate groups). [file 12889_2020_9555_MOESM7_ESM.docx]

**TOPIC GUIDE FOR FOCUS GROUP DISCUSSION BOYS AND GIRLS (IN SEPARATE GROUPS)**

Let the focus group participants discuss the topic with each other and make sure that you use this only as a true guide in the focus group discussion and not as a list of questions to be covered one after the other. Encourage all members to participate and do not let one person dominate. Before starting the discussion under each theme (A-F), please read the theme introduction and all questions sorting under that theme.

**A Introduction: A case**

Grace is a 16 years old school girl. She has just started form 2. She has a boyfriend who is 24 and now she has discovered that she is pregnant. Her parents are farmers and she has 5 siblings, 3 boys and 2 girls. Grace would like to continue her schooling, but now she does not know what to do.

How do you consider this situation? Is this something that could happen in this community?

What options does Grace have? What would you expect her to do? Would you encourage her to continue / resume school after childbirth? Would you expect her to marry?

If you were her parents, what would you do?

How will Grace be considered by her family, friends and community?

Why do you think Grace ended up being pregnant?

**B Pregnancy and childbirth**

We would now like you to discuss with each other in the group norms related to early pregnancy and childbirth.

When is a woman expected to have children in this community?

When and under what circumstances is pregnancy unacceptable or unwanted?

Where do girls and boys get information about sexual and reproductive health?

How is access to condoms and contraceptives?

**C Marriage**

Now we are moving into the issue of marriage and would like you to discuss norms and values attached to marriage in your age group and in your community.

What do you think is the best time for marriage? Why is that so?

How common is teenage marriage? How do you consider marriage below 18?

How would the parents and the community react if a girl got married while still in school or if she stopped school to get married?

Who decides on marriage? Can a girl decide herself when and whom to marry?

**D Education**

Education is

How do parents in this area value schooling/education? Is the importance of education different for boys and girls? How many years in school is desirable for boys and girls?

How do you think the present school system prepares the pupils for the life ahead?

Is school drop-out a problem in this area? If so, who drops out and why?

Would it be possible for a girl to re-enter school after childbirth?

**F Interventions**

What do you think would work to prevent girls from getting pregnant early?

What do you think would be the best strategies to reduce early marriage in this community?

What do you think would be the best strategies to retain girls in school and to encourage the education of girls in general?
