# Supplementary material for: Vanilla bisquits and lobola bridewealth: parallel discourses on early pregnancy and schooling in rural Zambia
Source: BMC Public Health. 2020 Oct 1;20:1485. doi: 10.1186/s12889-020-09555-y (PMC7528241; doi:10.1186/s12889-020-09555-y)
Supplement: Supplementary file 8 — Additional file 8. Topic guide ‘Adults’. [file 12889_2020_9555_MOESM8_ESM.docx]

**FOCUS GROUP DISCUSSION GUIDE FOR ADULT MEN AND WOMEN**

Let the focus group participants discuss the topic with each other and make sure that you use this only as a true guide in the focus group discussion and not as a list of questions to be covered one after the other. Encourage all members to participate and do not let one person dominate. Remember to follow up and probe interesting issues that come up in the discussion.

**A Introduction: A case**

Grace is a 16 years old school girl. She has just started form 2. She has a boyfriend who is 24 and now she has discovered that she is pregnant. Her parents are farmers and she has 5 siblings, 3 boys and 2 girls. Grace would like to continue her schooling, but now she does not know what to do.

How do you consider this situation? Is this something that could happen in this community?

What options does Grace have? What would you expect her to do? Would you encourage her to continue / resume school after childbirth? Would you expect her to marry?

If you were her parents, what would you do?

How will Grace be considered by her family, friends and community?

Why do you think Grace ended up being pregnant?

**B Pregnancy and childbirth**

How common is pregnancy among teenagers in this community?

Is it considered a problem? At what age is it considered a problem?

When is a woman expected to have children in this community?

When and under what circumstances is pregnancy unacceptable or unwanted?

**C Marriage**

What do you think is the best time for marriage? Why is that so?

What do you think are the main reasons for early marriages in this community?

How would the parents and the community react if a girl stopped school to get married?

What role do you think parents/guardians and community cultural practices play in ‘early’ marriages in this community?

**D Education**

How do parents in this area value schooling/education? Is the importance of education different for boys and girls? How many years in school is desirable for boys and girls?

How do you think the present school system prepares the pupils for the life ahead?

Can education be a barrier to marriage/childbearing? Can marriage get in the way of schooling?

Is school drop-out a problem in this area? If so, who drops out and why?

Would it be possible for a girl to re-enter school after childbirth?

**F Interventions**

What do you think would work to prevent girls from getting pregnant early?

What do you think would be the best strategies to reduce early marriage in this community?

What do you think would be the best strategies to retain girls in school and to encourage the education of girls in general?
